# A Statistical Analysis of the Impact of Gun Ownership on Mass Shootings in the USA Between 2013 and 2022

**DOI:** 10.1007/s11524-024-00881-9

**Published:** 2024-06-03

**Authors:** Madison Daraklis, Mehul Pol, Lindsey Johnson, Cianna Salvatora, Lucy Kerns

**Affiliations:** 1 Department of Mathematics, Westminster College, New Wilmington, PA USA; 2 Department of Statistics, The University of Virginia, Charlottesville, VA USA; 3 Department of Mathematics, Wellesley College, Wellesley, MA USA; 4 Department of Mathematics & Statistics, Youngstown State University, One University Plaza, Youngstown, OH 44555 USA

**Keywords:** Mass shootings, Gun ownership, Negative binomial generalized linear mixed model, Mass shooting incidents, Mass shooting fatalities

## Abstract

Mass shootings (incidents with four or more people shot in a single event, not including the shooter) are becoming more frequent in the United States, posing a significant threat to public health and safety in the country. In the current study, we intended to analyze the impact of state-level prevalence of gun ownership on mass shootings—both the frequency and severity of these events. We applied the negative binomial generalized linear mixed model to investigate the association between gun ownership rate, as measured by a proxy (i.e., the proportion of suicides committed with firearms to total suicides), and population-adjusted rates of mass shooting incidents and fatalities at the state level from 2013 to 2022. Gun ownership was found to be significantly associated with the rate of mass shooting fatalities. Specifically, our model indicated that for every 1-SD increase—that is, for every 12.5% increase—in gun ownership, the rate of mass shooting fatalities increased by 34% (*p* value < 0.001). However, no significant association was found between gun ownership and rate of mass shooting incidents. These findings suggest that restricting gun ownership (and therefore reducing availability to guns) may not decrease the number of mass shooting events, but it may save lives when these events occur.

## Introduction

Gun violence represents a major issue within the United States (US). Among the types of gun violence in the US, mass shootings—both the number of incidents and fatalities—have risen sharply in the last decade, posing a significant threat to public health and safety in the country [1–3]. This upward trend is particularly troubling in recent years; for example, in 2021 alone, there have been 676 mass shooting incidents that have killed 694 people and injured 2774 others [4]. Compared with other developed countries, the US has substantially higher rates of mass shooting incidents, accounting for only 5% of the world’s population but 73% of global mass shootings that occurred over the last two decades, according to a study that compared mass shootings in the US against developed and developing countries between 1998 and 2019 [5]. Other research examining global mass shootings also suggests that mass shootings are a uniquely American problem, particularly in relation to other developed countries [6, 7].

While mass shootings are relatively rare compared with other forms of gun violence, they disproportionately affect society and influence policy as these attacks cause public fear and subsequent demands for policy change [8]. As news of mass shootings continues to plague American televisions, questions surrounding these tragedies remain unanswered—why is the rate of mass shootings increasing and how can we reverse the trend? In recent years, there has been a growing body of research examining mass shootings in the US in an effort to better understand the factors behind mass shootings and provide effective intervention strategies for preventing such violent acts. Although it is hard to find the exact root causes of mass shootings, prior research has identified several risk factors that might be contributing to this epidemic and its growing frequency, including gun policies [9–15], mental health [8, 16–18], and media coverage [8, 19, 20].

In addition to the aforementioned risk factors, the prevalence of gun ownership has also received considerable attention in connection with mass shootings. In fact, imposing restrictions on gun ownership—that is, limiting access to guns—has been frequently seen as a common response for dealing with mass shootings throughout the world. The US has higher rates of gun ownership than do other countries. Previous studies comparing mass shootings in the US against other countries showed that US mass shootings were more likely to involve multiple firearms than all other countries [5, 6]. This higher chance of US shooters arming themselves with more than one firearm may be partially attributable to the ease of gun accessibility in America [18]. According to a 2018 report from the Small Arms Survey—an independent Swiss-based research project—US civilians had the highest rate of gun ownership in the world, with an estimated 120.5 firearms per 100 residents, followed distantly by Yemen at 52.8 firearms per 100 residents [21]. For further reference, Canada, a close neighbor of the United States, has an estimated 34.7 guns per 100 residents. More recent data suggest that gun ownership grew much more rapidly over the last few years. For example, according to Small Arms Analytics, a consulting firm that tracks gun sales, US gun sales reached a record 23 million in 2020—which is a 65% increase from 2019—and it remained high in 2021 [22]. This acceleration in firearms purchases in the US was also reported by a study in 2022, which found that 1.5% of US adults (approximately 3.8 million) became new gun owners—an increase of 1.4 million compared with the 0.9% of US adults who did so in 2019—and overall 2.9% of US adults (7.5 million) became new gun owners between January 2019 and April 2021 [23].

Gun control is a highly controversial topic in the US that, for decades, has generated heated discussion about issues such as public safety, individual rights, and government oversight. In recent years, the surge in mass shootings and extensive media coverage on high-profile public mass shootings has intensified the debate over gun control, with much of the argument centering on whether restricting gun ownership (and therefore reducing access to guns) would help reduce mass shootings and gun violence. Clearly, understanding the relationship between the prevalence of gun ownership and mass shootings is critical to addressing these violent crimes and guiding public health decisions regarding prevention strategies. There have been research efforts directed at exploring the relationship between firearm prevalence and firearm-related violence. However, most of these studies were primarily concerned with the association between the prevalence of gun ownership and homicide rates [24–30], or the association between the prevalence of gun ownership and suicide rates [31–34].

Fewer studies have specifically focused on mass shootings and the effects of gun ownership rates on this phenomenon, and the results were mixed. A cross-national study conducted by Lemieux in 2014, in which mass shootings between 25 developed countries were compared, showed that there was a strong positive correlation between the gun ownership rate and the number of mass shootings and related casualties [7]. Another cross-national study was conducted two years later by Lankford, which looked at public mass shootings in 171 countries from 1966 to 2012, and examined the effects of gun ownership rates, along with other factors, on public mass shootings. The results suggested that public mass shootings were partially attributable to the cross-national differences in gun availability [35]. Reeping et al. used state-level data for mass shooting incidents and gun ownership rates in the US between 1998 and 2015 and found that states with higher levels of gun ownership had higher rates of mass shooting incidents [9]. Fridel also examined the impact of gun ownership on counts of mass shootings at the state level in the US, and reported similar findings that mass shootings disproportionately occurred in states with higher levels of gun ownership [36]. More recent work by Reeping et al. focused on K-12 school mass shootings and found that higher rates of state-level gun ownership were associated with higher rates of these incidents [37]. However, Lin et al. used the mass shooting data in the US from 1982 and 2018 to evaluate if some state-level risk factors—including gun ownership rate—could predict the state-level mass shooting rate, and they did not find a correlation between the rates of mass shootings and gun ownership [8]. Another study by Nugent et al. also showed that there was no evidence of a relationship between mass shootings and gun ownership at the state level [38].

In spite of the increasing prevalence of mass shootings in the US and intense media attention these tragedies have received, the definition of a mass shooting is neither agreed upon nor standardized. The Federal Bureau of Investigation (FBI) defines a mass shooting as a single event in which four or more people are killed, excluding the shooter, and the FBI Supplementary Homicide Report provides mass shooting data that are collected based on this definition [39]. However, other databases have collected data using different definitions; for example, the Gun Violence Archive (GVA) defines a mass shooting as an incidence of gun violence in which four or more people are killed or injured, excluding the shooter [4], and the Mass Shooting Tracker set a criterion of four or more people injured or killed, including the shooter [40].

In this study, we used the GVA mass shooting data from 2013 to 2022 to explore trends in mass shootings over this 10-year period and examine differences in gun ownership rate among the 50 states. We then sought to examine how state gun ownership rate affects mass shooting events; specifically, we conducted cross-state time series analyses to assess the impact of state gun ownership on rates of population-adjusted mass shooting incidents and related fatalities.

## Methods

### Data Sources

For our research, we used the GVA’s definition of a mass shooting and collected mass shooting data that occurred between 2013 and 2022 from its database. The GVA, established in 2013, is an independent and non-profit organization formed to provide free online data about gun violence in the US. It provides comprehensive information about gun violence incidents, including time and location of incidents, information about victims and perpetrators such as age and gender, type of guns used, etc.

Because gun ownership is not directly surveyed in all 50 states each year in the US, we used a proxy measure, the proportion of suicides by firearms to total suicides (often denoted as FS/S), that has been shown to be highly correlated with direct survey measures of household gun prevalence [41, 42]. This measure has long been regarded as a reliable indicator of US household with at least one gun and has been extensively used as a proxy for state gun ownership in literature [8, 9, 11, 43, 44]. We obtained the data on firearm-related suicides and all suicides from the Centers for Disease Control and Prevention’s Web-Based Injury Statistics Query and Reporting Systems (WISQARS) database [45].

We included year as a covariate in all analyses to account for time-specific effects, and we also considered some state-level socio-economic variables that are often used in gun violence literature, including median income, the percentage of people living below poverty, unemployment rate, the percentage of people with a high school diploma or equivalent, the percentage of young adults aged 18–24 years, the percentage of Black people, incarceration rate, and law enforcement officer rate. All socio-economic data were gathered from the American Community Survey at the US Census Bureau [46], except for the incarceration rate and law enforcement officer rate, which were obtained from the Bureau of Justice Statistics and the US FBI Uniform Crime Report, respectively [47, 48]. We further retrieved the state population data from the US Census Bureau to account for population differences among the states [49].

The state-level suicide and socio-economic data were not available for 2022, so we used a 3-year weighted moving average approach with weights 1/6, 2/6, and 3/6 for the years 2019–2021, respectively, to estimate these missing values.

### Statistical Methods

To understand how the rate of mass shooting incidents varied from state to state, we first computed the population-based mass shooting rate in each of the 50 states across all years from 2013 to 2022. We then calculated the annual counts of mass shooting incidents and fatalities in the US to assess the temporal trend of the mass shootings over the study period. A bar plot and a box plot were constructed to display information on state-level gun prevalence, represented by the average annual rate of gun ownership per state over the study period. We also constructed scatterplots between the state-level prevalence of gun ownership and rates of mass shooting incidents and fatalities across all states in all years.

To investigate the association between state gun ownership rate and rates of mass shootings incidents and fatalities, we employed a generalized linear mixed model with a negative binomial distribution (negative binomial GLMM), which accounts for among-state variation in counts of mass shooting incidents and fatalities as well as variation within states. We chose to model both count data (i.e., number of mass shooting incidents and number of mortalities due to mass shootings) with a negative binomial distribution because overdispersion (the variance is greater than the mean) was present in both outcome variables. More specifically, we let *Y*_*it*_ represent the number of mass shooting incidents or fatalities at time *t* and state *i*, which is assumed to follow the negative binomial distribution:

1 $${Y}_{it}\sim NB\left({\mu}_{it,}\ {\mu}_{it}+\theta {\mu}_{it}^2\right)$$

where *μ*_*it*_ is the mean parameter (i.e., the average count of mass shooting incidents or fatalities at time *t* and state *i*) and *θ* is the dispersion parameter that controls the amount of dispersion. The mean *μ*_*it*_ is related to the fixed state-level effects and random state effect via the following logarithm link function:

2 $$\log \left({\mu}_{it}\right)=\log \left({P}_{it}\right)+{x}_{it}^{\prime}{\beta} +{b}_i$$

where *β* is the 1 × 11 vector of fixed effects (including the intercept *β*_0_) to represent the coefficients of the covariates *x*_*it*_, and *b*_*i*_ is the random state effect that is assumed to follow a normal distribution (i.e., *b*_*i*_~*N*(0, *σ*^2^)). The covariate vector *x*_*it*_ in our model includes the gun ownership proxy, year, and eight socio-economic covariates (that is, median income, the percentage of people living below poverty, unemployment rate, the percentage of people with a high school diploma or equivalent, the percentage of young adults aged 18–24 years, the percentage of Black people, incarceration rate, and law enforcement officer rate). We treated the logarithms of state-specific population size, log(*P*_*it*_), as an offset, to examine the relationship between covariates and rates of mass shooting incidents and fatalities (i.e., number of mass shooting incidents and fatalities per 100,000 residents) at the state level. Note that year was included as a covariate to represent the underlying annual trend. Also, the state-specific random effect *b*_*i*_ was added to model the dependence in counts of mass shooting incidents and fatalities within each state, since repeated counts were measured for the same state over time between 2013 and 2022.

To examine the relationship between gun prevalence and rates of mass shooting incidents and fatalities, while controlling for potential confounding variables, we first ran a full model with all covariates, as specified in Eqs. (1) and (2). We then ran a partially adjusted model by including the socio-economic covariates with coefficients in the full model that had *p* values less than 0.10, which is our final and more parsimonious model. Note that the partially adjusted models included the variables year and gun ownership proxy, but limited inclusion of less influential socio-economic covariates.

All data analyses were performed using R Statistical Software (version 4.2.2; R Core Team 2022) [50]. To fit the negative binomial GLMM, we applied the glmmTMB function from the “glmmTMB” package [51]. Since some of the covariates were measured on very different scales, which caused convergence issues when fitting the model, we standardized all covariates before including them in the model to improve the performance of the optimization algorithm used in the glmmTMB function. The significance level for all statistical hypothesis tests was set to be 0.05.

## Results

**Fig. 1:**
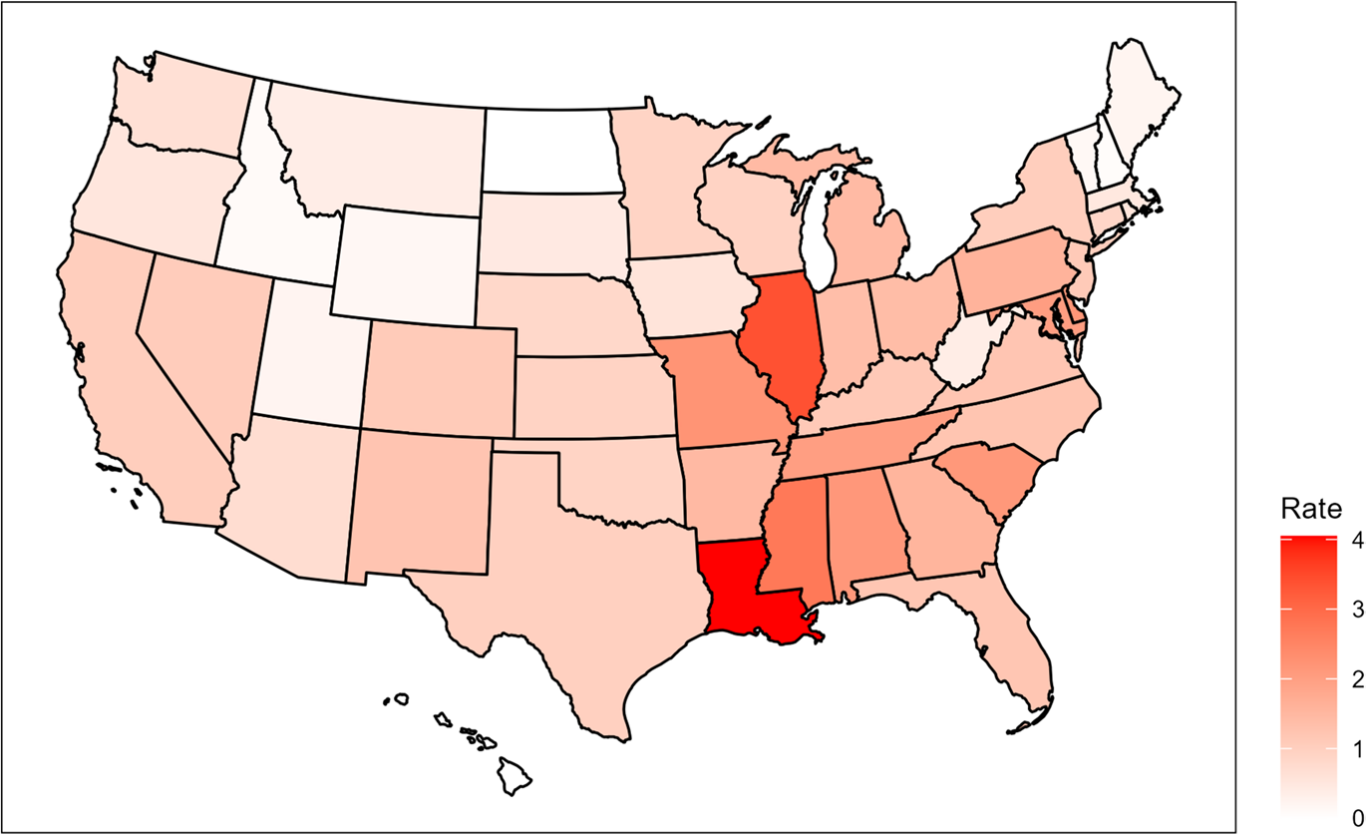
Number of mass shooting incidents per 100,000 residents for all US states between 2013 and 2022

Between January of 2013 and December of 2022, there were a total of 4216 mass shooting events recorded in the US (excluding the District of Columbia), with 4473 fatalities and 17,497 injuries. The heatmap in Fig. 1 displays the rate of mass shooting incidents—i.e., number of mass shooting events per 100,000 residents—for all US states from 2013 to 2022. Overall, it can be seen that states in the Southeast and Midwest regions had higher rates of mass shooting incidents than those in other regions in the country, particularly when compared with states in the Northwest and North central regions. Louisiana, Illinois, and Mississippi had the highest rate of mass shootings incidents, with 4.05, 3.38, and 2.72 mass shootings respectively per 100,000 residents over the 10-year study period. Neither North Dakota nor Hawaii reported any mass shootings during the given time period.

**Fig. 2:**
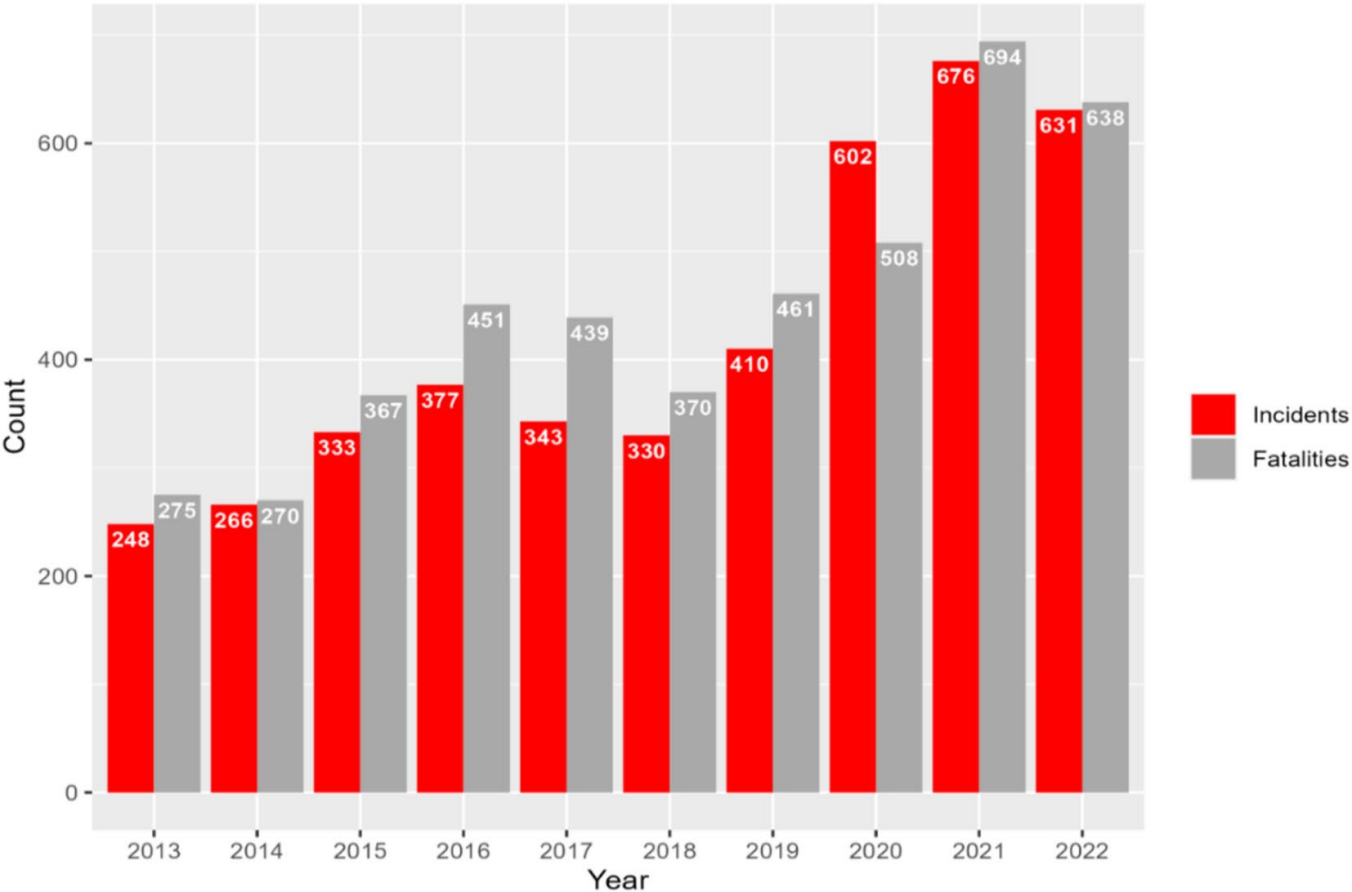
Number of mass shooting incidents and fatalities per year

Figure 2 shows the annual counts of mass shooting incidents and fatalities from 2013 to 2022. For the nation as a whole, an upward trend can be observed overall for the number of mass shooting incidents over the 10-year study period, with a 2.5-fold increase from 2013 (248 incidents) to 2022 (631 incidents). The most noticeable change is the sharp rise between 2019 (410 incidents) and 2020 (602 incidents)—a 31.9% increase in a single year. The inclines in the number of mass shooting incidents were accompanied by inclines in the number of fatalities caused by mass shootings, with the number having more than doubled from 275 in 2013 to 638 in 2022.

**Fig. 3:**
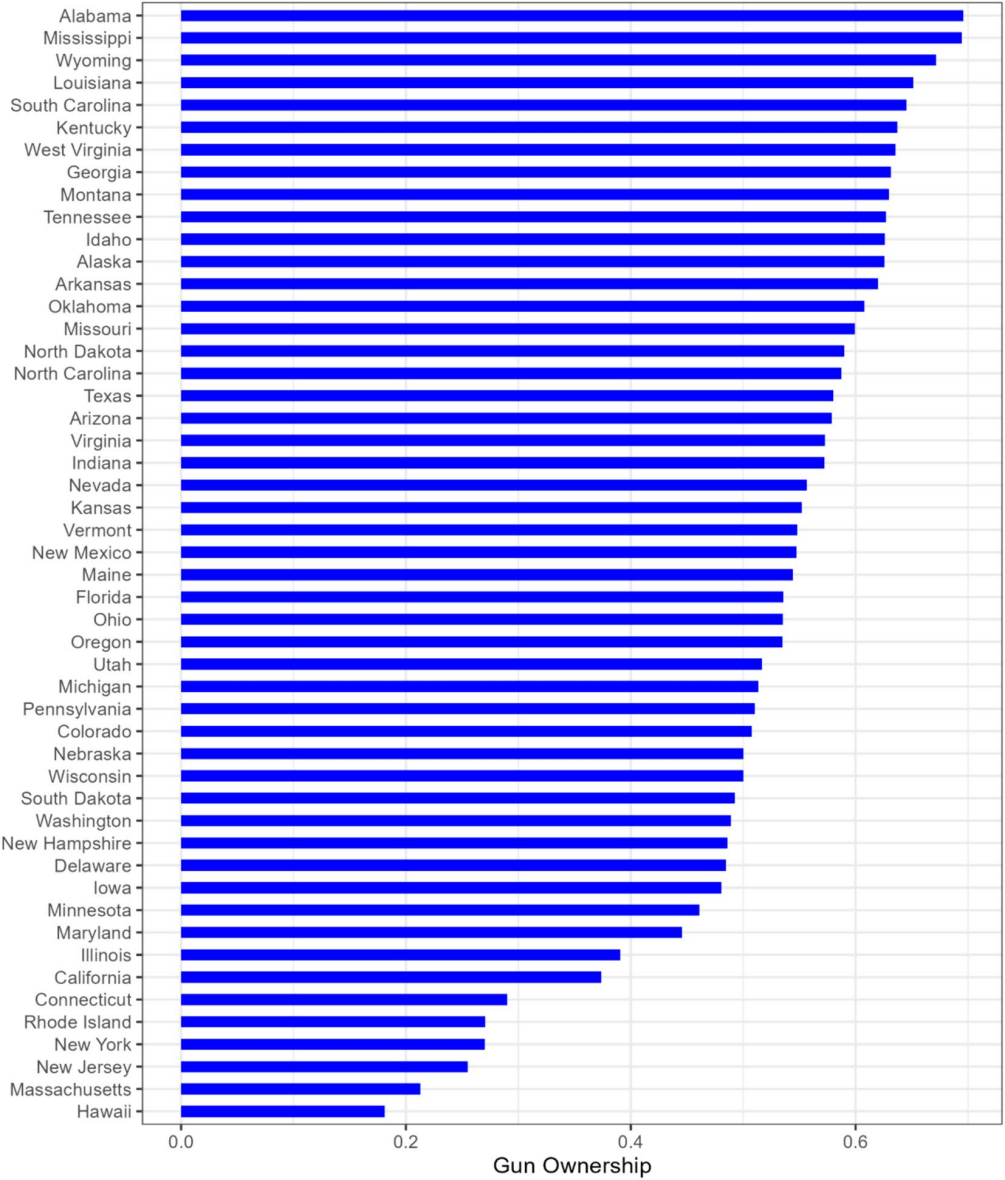
Average rate of gun ownership per state over study period (2013–2022)

**Fig. 4:**
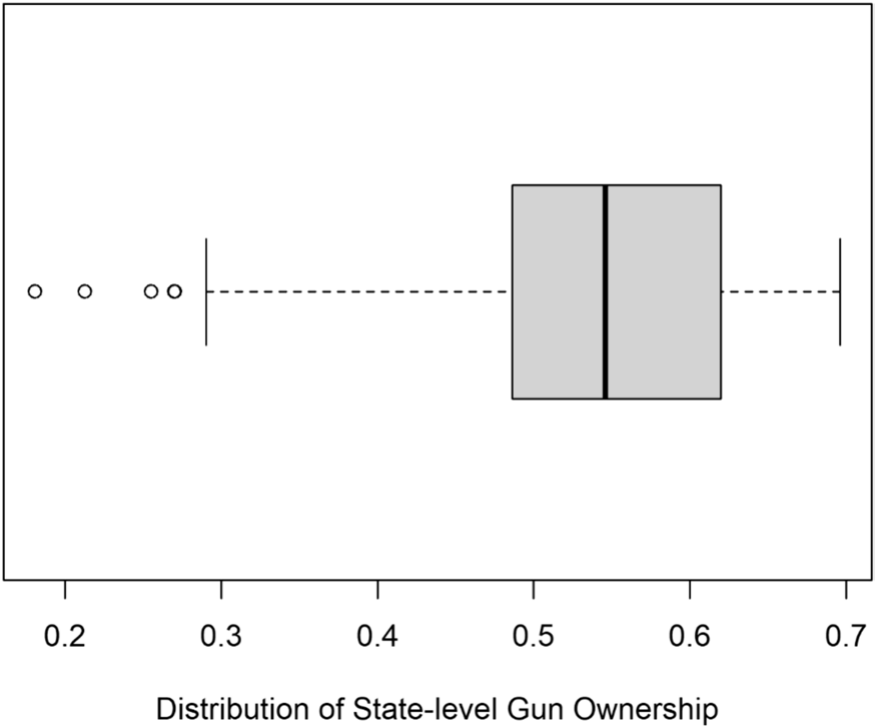
Box plot of average rate of gun ownership for all states from 2013 to 2022

Over the study period, the mean estimated rate of gun ownership (measured by the FS/S proxy) ranged from a low of 18.1% in Hawaii to a high of 69.6% in Alabama, with an average over all states of 52.1% (standard deviation (SD) = 12.5%). Figure 3 shows a bar plot that displays the average rate of gun ownership per state over the period. The box plot in Fig. 4 displays the distribution of the average gun ownership rate for the 50 states. It appears that the gun ownership rate has a left-skewed distribution in which more than half of the states had an average rate of gun ownership higher than the overall mean rate of gun ownership. The four states that were identified as outliers in the distribution with extremely small gun ownership rates are Hawaii (18.1%), Massachusetts (21.3%), New Jersey (25.5%), and New York (27.0%).

**Fig. 5:**
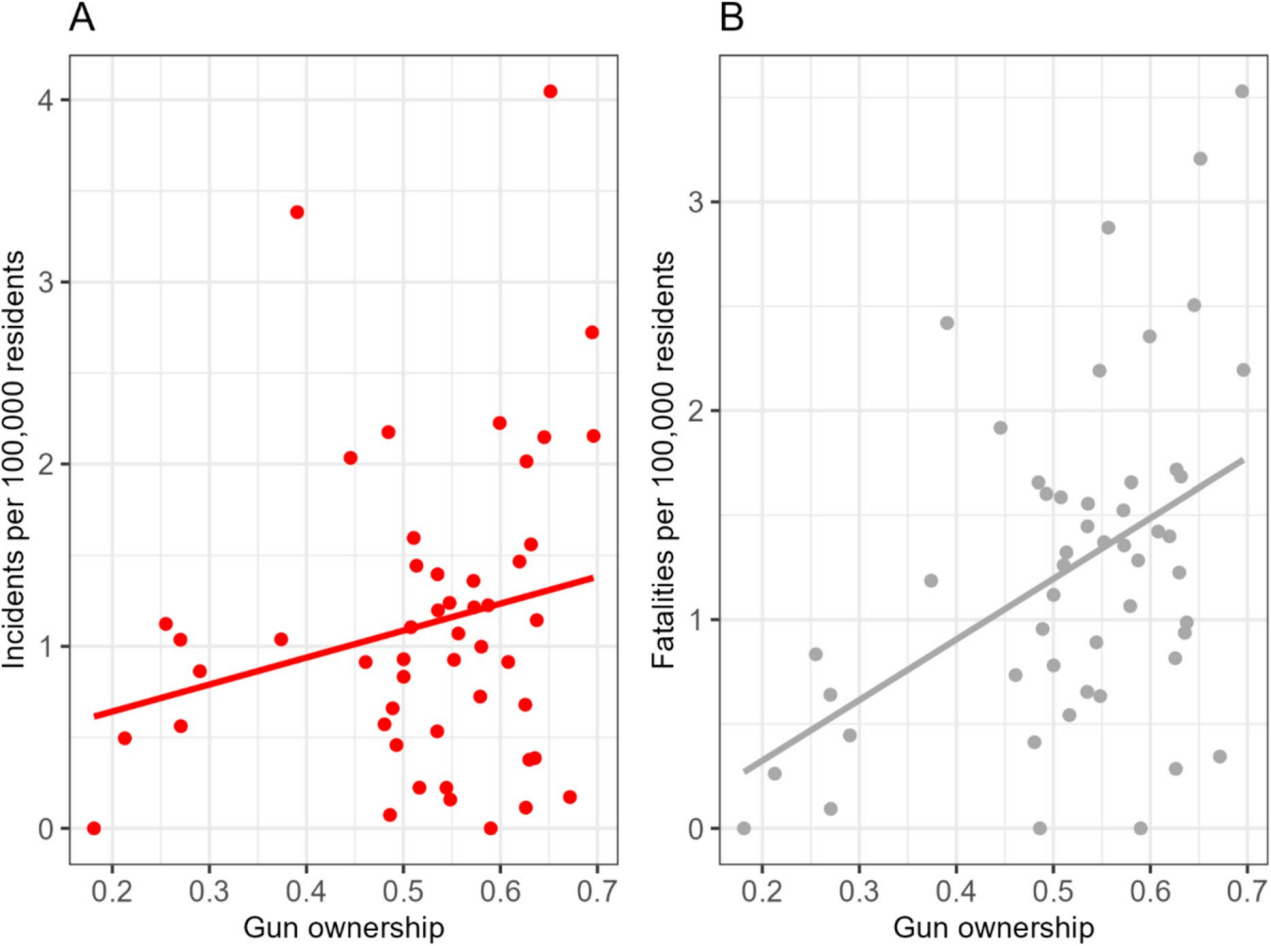
Scatterplots of the relations between gun ownership and rates of mass shooting incidents (**A**) and fatalities (**B**)

Figure 5 displays scatterplots of gun ownership and rates of mass shooting incidents and fatalities. While a general upward trend is seen in both scatterplots—that is, states with higher rates of gun ownership generally had higher rates of mass shooting incidents and fatalities—the positive correlation between the prevalence of gun ownership and rates of mass shooting fatalities appeared to be stronger than that between the prevalence of gun ownership and rates of mass shooting incidents.

In our final models, we transformed the estimated coefficients for the predictors into the incidence rate ratio (IRR) of mass shooting incidents and fatalities by an exponential function to ease interpretation of results. The negative binomial GLMM that examined the mass shooting incidence rate showed that there was no statistically significant correlation between the rate of mass shooting incidents and gun ownership (IRR = 1.06, 95% confidence interval = (0.90, 1.25), *p* value = 0.507), adjusted for the other predictors in the model. However, the results from the negative binomial GLMM with the number of mass shooting fatalities as the outcome variable revealed that gun ownership was a statistically significant predictor of mass shooting fatality rate. Table 1 displays the results from the partially adjusted model with the number of mass shooting fatalities as the outcome variable. As mentioned earlier, this parsimonious model includes only socio-economic covariates that had coefficients with *p* values less than 0.10, in addition to gun ownership and year.

**Table 1:** Summary of negative binomial GLMM for variables predicting mass shooting fatality rates

Variable	IRR (95% CI)	*p* value
Gun ownership	1.34 (1.16, 1.53)	< 0.001
Year	1.24 (1.14, 1.34)	< 0.001
Percent Black	1.37 (1.20, 1.58)	< 0.001

Note that the results in the table were calculated based on the standardized coefficients for the predictors in the partially adjusted model. So, the estimated IRR of 1.34 for the gun ownership indicates that for every 1-SD increase in the state gun ownership proxy, the rate of mass shooting fatalities is increased by 34% (IRR = 1.34, 95% confidence interval = (1.16, 1.53), *p* value < 0.001), controlling for other predictor variables in the model. In other words, a 12.5% increase in the gun ownership proxy (the SD of gun ownership is 12.5%) is associated with a 34% increase in the rate of mass shooting fatalities. Percent of Black population was also significantly associated with the rate of mass shooting fatalities (*p* value < 0.001). It had an estimated IRR of 1.37 (95% confidence interval = (1.20, 1.58)), indicating that a 9.4% increase in the percentage of Black population (the SD of percent of Black population is 9.4%) is associated with a 37% increase in the rate of mass shooting fatalities, controlling for other predictor variables. Additionally, the estimated IRR of 1.24 for the predictor, year, indicates a statistically significant time trend (IRR = 1.24, 95% confidence interval = (1.14, 1.34), *p* value < 0.001), after adjusting for other predictors in the model. For the random state effect, the variance was estimated to be ${\hat{\sigma}}^2=0.1428$. A likelihood ratio test was performed to assess the significance of the random effect. Results of the test *H*_0_ : *σ*^2^ = 0 revealed that the random-effect variance was significantly different from 0 (test statistic *χ*^2^ = 27.735, *p* value < 0.001 referenced against ${\chi}_1^2$), indicating that there is significant state-to-state variability in the rate of mass shooting fatalities.

## Discussion

We used a generalized linear mixed model with a negative binomial distribution to investigate how state-level gun ownership affects the occurrence of mass shootings and associated casualties. Our analyses show that the prevalence of gun ownership had statistically significant effects on the rate of mass shooting fatalities in the US and that states with higher rate of gun ownership had higher rates of mass shooting fatalities than states with lower gun ownership rate. However, the prevalence of gun ownership was not associated with the rate of mass shooting incidents.

Our findings that the rate of gun ownership was not significantly correlated with the rate of mass shooting incidents are consistent with those reported by Lin et al. and Nugent et al. [8, 38], but they conflict with results from some of the previous work on this topic [9, 36, 37]. The inconsistency in findings from different studies could be partly due to the different databases used by researchers to examine the mass shooting incidents. As mentioned earlier, no universal definition for mass shootings exists and different databases—e.g., FBI Supplementary Homicide Reports and Mass Shooting Tracker—use different definitions to track and collect these events. This lack of consistency between databases makes it very difficult to make meaningful comparisons between different studies on mass shootings, which can lead to misleading findings that could hinder our understanding of mass shooting events (such as motives, trends, risk factor, and impact of policy changes) and limit our ability to implement effective measures to address this public health issue. Therefore, it is highly recommended to have a standard definition for mass shootings to help ensure the comparability of research findings and a better understanding of this crisis.

The discrepancy in research findings could also be attributed to the fact that there is disagreement on whether to include non-public mass shootings that occur in connection with domestic violence or criminal activities (e.g., armed robbery and gang violence). Some sources (e.g., Mass Shooter Tracker) exclude non-public mass shooting incidents and count only indiscriminate incidents that occur in a relatively public place and in which victims appear to be selected randomly. The GVA data, on the other hand, count as mass shootings all incidents that satisfy its definition of four or more people shot in a single event, regardless of the perpetrator’s motivation to commit mass murder or the circumstances that surround the event. Given that mass shooting incidents vary greatly in circumstances and motivations, and that the determination of the nature of incidents can be subjective, this disagreement between data sources could lead to a substantial difference in which incidents are counted. For example, GVA recorded a total of 343 mass shooting incidents in 2017 whereas Mother Jones recorded only 11 incidents in the same year [52]. Such discrepancy will inevitably produce controversial conclusions in mass shooting studies using different sources, since statistics can be heavily influenced by the database from which they are generated.

The significant correlation between the rates of state gun ownership and mass shooting fatalities suggests that although restricting access to guns may not decrease the number of mass shooting events, it may save lives when these events occur (that is, it may decrease the number of deaths caused by mass shootings). To the best of our knowledge, there were no previous studies that focused specifically on the association between rates of state gun ownership and mass shooting fatalities in the US. However, prior research has shown that the number of deaths by mass shootings could be related to the gun ownership rate through a third variable. For example, the cross-national study conducted by Lemieux found that the gun ownership variable was the best predictor for death by firearms, which in turn was found to be significantly related to mass shooting casualties [7]. Other studies have found that more firearms used in mass shootings increased the number of fatalities during an attack [6, 53]. This finding also suggests that the rate of gun ownership may affect mass shooting fatalities, as higher gun ownership rates tend to increase the chance of mass shooting perpetrators using more than one firearm in an assault, leading to increased risks of fatal mass shootings.

We found that the percent of Black population was a significant predictor of both mass shooting fatalities and incidents (*p* value < 0.001 in the partially adjusted model with the number of mass shooting incidents as the outcome variable), and that states with higher proportions of African Americans are more likely to experience mass shootings. One implication of this result is that gun violence has a disproportionate impact on Black Americans as they suffer higher rates of gun violence compared to other racial groups, a finding observed in previous research examining racial disparities in firearm-related crimes [54, 55]. These racial disparities often result from a systematic and structural disadvantage of the Black population, including racial inequities such as unequal access to housing, education and employment opportunities, and historical disinvestment in public infrastructure and services in the Black community. As such, community-based violence intervention programs for addressing root causes of gun violence, coupled with evidence-based gun safety laws, are important tools to reduce racial inequality in gun violence victimization rates.

### Limitations

There are several limitations within the current research. One is that direct measurement of state-level gun ownership was not available, and we relied on a proxy measure (i.e., the proportion of suicides committed with a firearm). Despite its wide use, the accuracy and validity of this proxy measure have been questioned by some researchers. For example, Kleck pointed out that the validity of this measure established in previous cross-sectional research does not guarantee its adequate validity in research with a longitudinal design [56]. It is also worth noting that a recent article examining four proxy measures for county-level gun ownership including the FS/S found that the per capita rate of federal firearm licenses was the best proxy measure for gun ownership at the county level [57]. Alternative proxies have been proposed in an effort to better estimate state gun ownership [58, 59], but more research is needed to validate these new measures. As a result, research studies concerning the effects of firearm prevalence on firearm violence such as ours have been impeded by the lack of direct measures of gun ownership and the uncertainty regarding the validity of proxy measures.

Another limitation is that although our study revealed that the rate of mass shooting fatalities was highly correlated with the prevalence of gun ownership, this association could have been confounded by some unaccounted factors, such as overall strength of state gun laws, media coverage of mass shooting events, and other socio-economic factors (e.g., percentage of female headed households, percent population identified as male, and percent military veteran). In addition, our study did not provide information about a cause-and-effect relationship. One may consider a lagged variable for gun ownership (e.g., gun ownership lagged by 1 or 2 years) to evaluate a causal relationship. Furthermore, there are concerns about a potential reverse causal association between gun ownership and mass shootings; that is, more mass shootings may motivate people to purchase guns for protection, and hence increase the level of gun ownership. Further research is encouraged to address these issues of causal relationship and reverse causation between prevalence of gun ownership and mass shooting events or, more broadly, gun violence incidents the per capita rate of federal firearm licenses.

## Conclusions

The rate of gun ownership—as measured by the proportion of suicides committed with a firearm—is significantly associated with the rate of mass shooting fatalities (i.e., number of deaths due to mass shootings per 100,000 residents), but not with the rate of mass shooting incidents at the state level in the US. Developing criteria to facilitate implementing a standardized definition of mass shootings across all databases is an important first step to advancing mass shooting research and prevention efforts. Also, having reliable data on gun ownership is essential in studies of firearm-related violence, particularly in the absence of direction measurement on this variable; therefore, more effort is warranted to develop and evaluate proxy measures that are accurate representations of the actual level of gun ownership.

## Data Availability

Not applicable, since the data used in the study are freely available to the public.
